# Theoretical explanation of rotational flow in the liquid-film motor

**DOI:** 10.1038/s41598-021-02470-1

**Published:** 2021-11-29

**Authors:** Ali Najafi, Reza Shirsavar

**Affiliations:** 1 Department of Physics, Institute for Advanced Studies in Basic Sciences (IASBS), Zanjan, 45137-66731 Iran; 2 Research Center for Basic Sciences and Modern Technologies (RBST), Institute for Advanced Studies in Basic Sciences, Zanjan, Iran; 3 Department of Physics, University of Zanjan, Zanjan, 45371-38791 Iran

**Keywords:** Fluid dynamics, Mechanical engineering

## Abstract

A liquid film that is under the action of two electric forces, an external electric field parallel to the film and a lateral voltage difference applied to both edges of the film, exhibits a universal rotational flow. In this article, we revisit this phenomena by considering an idealized so-called liquid-film motor and provide a theoretical description of the underlying physical mechanism that is responsible for the rotation. Based on this theory, the external electric field induces a non-uniform distribution of freely moving charges on the film. Then the internal field that is mainly resulted from the lateral voltage difference, will exert forces on induced charges and subsequently will result the rotational flow. We show, how the fields contribute in developing a universal flow pattern.

## Introduction

Fluid motion and flow pumping in small scale world, either in synthetic micro-fluidic devises or aqueous biological environments, have attracted many interests recent years^1,2^. Among very diverse proposed methods for inducing flow in small scale, the so-called liquid film motor is one of the most interesting ones^3,4^. In these experiments, it is shown that as a result of two externally applied electric forces, a controllable rotational flow can be produced in a suspended liquid film. A wide range range of isotropic fluids with different electric permittivities and dynamical properties like viscosity and surface tension have been used to see the rotation.

It should be mentioned that such vortex like patterns have been observed in freely suspended films of both Nematic^5^ and Smectic liquid crystals^6–8^. It is shown that the vortex patterns in such liquid crystal experiments can be well studied in the frame work of electro-convection based instability theory^9^. In addition to dielectric liquids, recent experiments showed interesting dynamical phenomena in fully conducting layer or droplet of metallic fluids where by application of electric field the metallic layer can start to move and a range of patterns will form^10,11^. In such experiments, the electrostatic mediated changes in surface tension and corresponding electro-capillary effects are responsible for the motion.

In spite of different experiments that support the idea of liquid film motors, there are some theoretical descriptions that consider different physical mechanisms for the observed rotation. Charge induction mechanism studied in two-dimensional geometry^12,13^ and three-dimensional geometry^14^ are among the proposed methods. In these works, numerical analysis are used to demonstrate the idea of charge induction mechanism.

Our goal in this article is to provide a theoretucal electro-kinetic based description that can capture the physics behind the observed rotational flow. Our description has common features with previously studied charge induction mechanism. We consider that the fluid motion is driven by electric forces in the medium where, the electric fields can simultaneously separate electric charges and enforce them to move. To develop a theoretical framework, we notice that in all experiments, the fluid is almost an electrically neutral solution that usually contains a kind of salt molecules. Ionization of such salt molecules, provides a number of freely moving electric charges in the liquid film. Depending on the experiment, ionic surfactant could be considered as another source of free charges in the system. Even pure water when interacting with atmospheric $\text{CO}_2$, could have a slight degree of acidity with carbonic acid which adds hydronium and bicarbonate ions to the fluid. For simplicity, we will only consider the contribution from the salt molecules and other types of ions can be easily added to the theory with no complexity.

In the following sections, we first introduce the model then we will provide the dynamical equations. Analytic solution for a very thin film and discussion about the role of physical characteristics of the fluid, will be presented at the two final sections.

## Model

**Figure 1:**
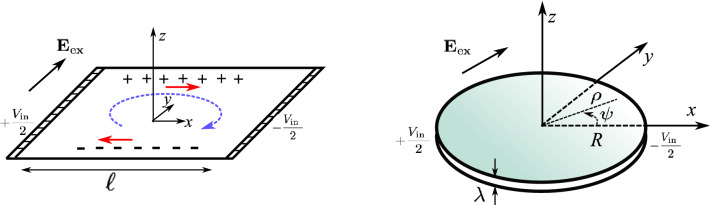
Left: schematic view of a liquid-film motor. A suspended liquid film is under the action of two electrical forcing. The first field is due to a voltage difference $V_{\text{ in }}$ that is applied to the conducting electrodes forming two lateral edges of the frame. The second force comes from an externally applied electric field $\mathbf{E}_{\text{ex}}$, asymptotically parallel to the plane of the film. As a result of these applied fields, the liquid fluid will eventually flow rotationally. Right: An idealized symmetric geometry where the frame is replaced by a circular frame with radius *R*. In this case a potential distribution $V_{\text{in}}(\psi )=-\frac{V_{\text{in}}}{2}\cos \psi$ is applied to the frame

Figure 1(left) shows the geometry of original liquid-film experiment where, a water film is suspended horizontally from a square-shaped frame. We denote by $\ell$ and $\lambda$, width and thickness of the film, respectively. Two lateral parts of the frame are conducting electrodes those are subjected to an electric potential difference $V_{\text{ in }}$. In addition to this electric potential, an external electric field $\mathbf{E}_{\text{ex}}$, that is initially uniform and parallel to the film, is applied to the system. In a reference frame that is shown in Fig. 1(left), the liquid film is located at $z=0$, the external field is given by $\mathbf{E}_{\text{ex}}={E}_{\text{ex}}{\hat{y}}$ and the edges of the frame at $x=\pm \ell /2$ are conducting electrodes. Emergence of a rotational flow in a universal direction given by $\mathbf{E}_{\text{ex}}\times {\hat{x}}$, is the main result of this experiment.

Here, we aim to provide a physical explanation of the details have been observed in the liquid-film motor. We present a minimal 2-dimensional model that is based on electro convection theory^15^ and it takes into account the physical characteristics of the film in the limit of $\lambda \ll \ell$. The film thickness $\lambda$, enters into our 2-dimensional model as a parameter that relates the physical parameters of our 2-dimensional model, like density and viscosity, to the bulk properties of liquid.

We simplify the geometry and replace the squared shape frame by a simple and idealized circular electrode (frame) having radius *R*. Figure 1(right), shows this geometry. The electrodes are denoted by a boundary potential, that is given by:

1 $$\begin{aligned} {V_{\text{in}}}(\psi )=-\frac{{V_{\text{in}}}}{2} \cos {\psi}, \end{aligned}$$

where $\psi$ is the azimuthal angle, as it is shown in the figure. We believe that this simplification does not change the main features of the real experiments but it will allow us to obtain analytical results for the potential distributions and it helps us to understand the underlying mechanism responsible for the fluid motion.

## Dynamical equations

Denoting the 2-dimensional flow and pressure field of the fluid by $\mathbf{u}$ and *P*, the dynamics of the incompressible fluid at steady state, is governed by the following equations:

2 $$\begin{aligned} \lambda \rho _{}(\mathbf{u}\cdot {\nabla }_{\text{s}})\mathbf{u}=-\nabla _{\text{s}} P+\lambda \eta _{}\nabla _{\text{s}}^2\mathbf{u}+ \mathbf{f}_{\text{b}},\,\,\,\mathbf{f}_{\text{b}}=-q\nabla _{\text{s}}\phi _{\text{s}},\,\,\, {\nabla }_{\text{s}}\cdot \mathbf{u}=0, \,\,\,\mathbf{u}|_{\text{frame}}=0, \end{aligned}$$

where $\eta$ and $\rho$ denote the viscosity and mass density (mass per unit volume) of a bulk liquid that the film is extracted from and ${\nabla }_{\text{s}}$ stands for the in-plane projection of the gradient operator. In the above dynamical equations, the electric body force $\mathbf{f}_{\text{b}}$ (force per unit area with in-plane components), is written in terms of surface charge density $q=q^++q^-$ (charge per unit area) and surface electric potential $\phi _{\text{s}}$. Here the surface charge densities of positive and negative charges are denoted by $q^+>0$ and $q^-<0$, respectively. Furthermore, the fluid flow is subjected to no-slip boundary condition at the edge of the solid frame.

It is worth mentioning that the role of surface tension is to compensate the gravity and it stabilizes the horizontal configuration of this liquid film. For a non-fluctuating and flat film, surface tension does not contribute to the fluid motion and it is ignored in the above dynamical equations.

To determine the electric body force, A full knowledge about the electric potential distribution on and in the vicinity of liquid film is necessary. As there is no free charges at air, the electric potential $\phi$ satisfies a 3-dimensional Laplace equation. This potential is subjected to boundary conditions on the electrodes, on the surface of film and at infinity as:

3 $$\begin{aligned} \nabla ^2\phi =0,\,\,\,\phi |_{\text{}\infty }=-E_{\text{ex}}y,\,\,\,\phi (\rho \le R,\psi ,z=0)=\phi _{\text{s}}(\rho ,\psi ),\,\,\,\phi _{\text {s}}(\rho =R,\psi )=V_{\text {in}}(\psi ), \end{aligned}$$

where $$\rho =\sqrt{x^2+y^2}$$.

Assuming that the surface potential $\phi _s(\rho ,\psi )$ is a known function, the above problem is a Dirichlet boundary value problem and it will provide a unique solution for $\phi (\rho ,\psi ,z)$. If we know the potential, we can obtain the charge distribution on the surface:

4 $$\begin{aligned} {q}{}=\epsilon _0\left( \partial _z\phi |_{z=0^+}-\partial _z\phi |_{z=0^-}\right) , \end{aligned}$$

where $\epsilon _0$ denotes the vacuum permitivity.

Following this strategy in solving the equations, the surface potential is a key element that is still unknown variable. To provide information about the surface potential and close the governing equations, we note that at steady state, surface electric current densities associated with positive and negative charges satisfy the continuity equations as $\nabla _{\text {s}}\cdot \mathbf{J}^{\pm }_{\text {s}}=0$. Denoting by $\mu$, the hydrodynamic mobility of charges, and considering the hydrodynamic convection and thermal diffusion of charges, we can write the overall current densities (for both positive and negative charges) as:

5 $$\begin{aligned} \mathbf{J}^{\pm }_{\text {s}}= \mp zQ_0 \mu q^\pm \nabla _{\text {s}}\phi _{\text {s}}+q^\pm \mathbf{u}-D\nabla _{\text {s}}q^\pm . \end{aligned}$$

One should note that, the electric current in this system is assumed to be conducted by ions. Electric conduction with small charges like electrons and protons, is neglected. Valence of ionic species (both positive and negative) is denoted by *z* and $Q_0=1.6\times 10^{-19}\text {C}$ stands for the fundamental charge. Furthermore, for simplicity we assumed that all charges have similar diffusion coefficient given by *D*. Additionally, we will assume that the system is very near to thermal equilibrium so that, we put $D=\mu k_BT$. For spherical ions with size *a*, hydrodynamic mobility is given by: $\mu =(6\pi \eta {a})^{-1}$. Electrolysis near the electrodes is an inevitable fact that can change the above scenario. Considering this effect, one needs to take into account the local distortions of the flow near electrodes. But, here for simplicity we have neglected this effect.

Taking into account the incompressibility condition, we can arrive at the following equation for surface charges:

6 $$\begin{aligned} (\mathbf{u}\cdot {\nabla }_{\text {s}})q^\pm =\mu \left( \pm zQ_0 \nabla _{\text {s}}\cdot \left( q^\pm \nabla _{\text {s}}\phi _{\text {s}}\right) + k_BT\nabla ^{2}_{\text {s}}q^\pm \right) , \end{aligned}$$

where, the left-hand side corresponds to the convection and the terms in the right-hand side, correspond to drift (electric conduction) and diffusion currents, respectively.

Equations (3), (4) and (6), form a complete set of strongly coupled equations that can capture the electrostatic variables, $\phi ,\,\phi _{\text {s}}$ and *q*, in our system. Using these fields, we can obtain the body force $\mathbf{f}_{\text {b}}$ and insert it in Eq. (2) to finally obtain the flow pattern in the system.

As a very important assumption in the above formulation, we have neglected the charge exchange on the electrodes. This is an experimental fact that the current appears in the circuit which provides the voltage difference, has a typical value of $\mu \text {A}$. This negligibly small current observed in the circuit, can be assigned to possible charge exchange mechanisms on the electrodes that we have neglected.

Before proceeding further, we need to examine the relative importance of different transport terms. For a typical experiment we have^4^:

7 $$\begin{aligned}&E_{\text{ex}}\sim 10^5\frac{\text{V}}{\hbox{m}},\,\,\,V_{\text{in}}\sim 10\,\text{V},\,\,\,R\sim \ell \sim 10\,\text{mm},\,\,\, u\sim 10^{-2}\frac{\text{m}}{\hbox{s}}\\&\lambda \sim 10^{-6}\text{m},\,\,\,\eta \sim 10^{-3}\text{Pa}\,\text{s},\,\,\,\mu \sim 10^{11}\frac{\text{m}}{\hbox{Ns}},\,\,\,k_BT\sim 10^{-21}\text{J}. \end{aligned}$$

Therefore, we can define and estimate two dimensionless quantities $\alpha$ and $\beta$, as:

8 $$\begin{aligned}&\alpha =\frac{\text{conduction}}{\hbox{convection}}\sim \frac{[Q_0\mu \nabla \phi _\text {s}]}{[u]}\sim 10^{1},\,\,\,\, \beta = \frac{\text{conduction}}{\hbox{diffusion}}\sim \frac{[Q_0\phi _\text{s}]}{[k_BT]}\sim 10^{5}, \end{aligned}$$

where, we have assumed that the electric field inside the film, has the same order of magnitude as the electric field in the air. As it is reflected from this dimensional analysis, the conduction term is the most important term in charge transport in the film.

It is instructive to have an estimation of the Reynolds number $\text{Re}$, that is defined as the ratio of inertial effects to dissipative forces. Regarding the typical values of the parameters, we will have:

$$\begin{aligned} \text{Re}=\frac{\text{inertia}}{\hbox{dissipation}}\sim \frac{[\rho \ell u]}{[\eta ]}\sim 10^{1}. \end{aligned}$$

One should note that the length in which the fluid velocity varies, is replaced by $\ell$, not $\lambda$. This is due to the experimental fact that no velocity gradient in film thickness has been observed. This result shows that both inertia and viscosity may play role in stabilizing a steady state profile for the flow pattern in this system.

## Conduction dominated regime

Neglecting the effects of convection and diffusion and keeping only the conduction, we can present a simplified picture that can reveal the underlying physical mechanism responsible for fluid motion. In this case, the dynamical equations (Eqs. 3, 4 and 6) read as:

9 $$\begin{aligned} \nabla ^2\phi ^{}=0,\,\,\,q=\epsilon _0\left( \partial _z\phi ^{}|_{z=0^+}-\partial _z\phi ^{}|_{z=0^-}\right) ,\,\,\, \nabla _{\text {s}} q^{}\cdot \nabla _{\text {s}}\phi ^{}_{\text {s}}=-q \nabla _{\text {s}}^{2}\phi ^{}_{\text {s}}, \end{aligned}$$

where $q=q^++q^-$ denotes the net charge. In terms of $\phi$ and *q*, first two equations of the above set, are linear but, as it is reflected from the last equation, a nonlinear relation between charge density and surface potential is expected. At the following part and to solve the equations, we choose a strategy that benefits the partial linearity of the equations.

Assuming that the surface potential is a known variable and using the linearity of Laplace equation, we can decompose the potential and charge density into two parts as: $\phi =\phi ^{I}+\phi ^{II}$ and $q=q^{I}+q^{II}$, respectively. These two fields satisfy the Laplace equations with the following boundary conditions:

10 $$\begin{aligned}&\phi ^{I}_{}|_{\infty }=-E_{\text {ex}}y,\,\,\,\phi ^{I}_{}|_{\text {on the film}}=0,\nonumber \\&\phi ^{II}_{}|_{\infty }=0,\,\,\,\,\,\,\,\,\phi ^{II}_{}|_{\text {on the film}}=\phi _{\text {s}}^{}(\rho ,\psi ). \end{aligned}$$

Now, we can present analytic results for fields $\phi ^{I}$ and $\phi ^{II}$.

Solution to the Laplace equation for $\phi ^{I}$ can be obtained in the oblate spheroidal coordinate system^16^. Oblate spheroidal coordinate system is defined by variables $(\xi ,\zeta ,\psi )$. Here $0\le \psi \le 2\pi$ measures the azimuthal angle around *z*-axis. Different values of $\xi$ construct a family of confocal oblate spheroids having their geometric center at the origin and different values of $\zeta$ show a family of confocal hyperboloids. Defining $\rho =\sqrt{x^2+y^2}$, the oblate spheroidal and the Cartesian coordinates are related as:

$$\begin{aligned} z=R\sinh \xi \cos \zeta ,\,\,\,\rho =R\cosh \xi \sin \zeta ,\,\,\,\tan \psi =\frac{y}{x},\,\,0\le \xi \le \infty ,\,\,0\le \zeta \le \pi . \end{aligned}$$

As one can see, $\xi =0$ defines a circular disk in $x-y$ plane with radius *R* (liquid film in our problem). As a result of separability of the Laplace equation in the oblate coordinate, the electric potential will read as:

11 $$\begin{aligned} \phi ^{I}_{}(\rho ,\xi )=-\frac{2}{\pi }\rho E_{\text {ex}}\left( \tan ^{-1}(\sinh \xi )+\frac{\sinh \xi }{1+\cosh ^2\xi } \right) \sin \psi , \end{aligned}$$

this potential corresponds to an induced surface charge on the disk that is given by:

12 $$\begin{aligned} q^{I}_{}(\rho ,\psi )=\frac{8\epsilon _0}{\pi }E_{\text {ex}}\frac{\rho }{\sqrt{R^2-\rho ^2}}\sin \psi . \end{aligned}$$

As we see, charges are accumulated near the edges of the disk with strong divergence at $\rho =R$.

To solve the Laplace equation for $\phi ^{II}$, we note that this problem can be considered as a mixed boundary value problem where in part of the plane $z=0$ (inside a circle with radius *R*), the potential is given while in the other parts of the same plane, the electric field is given (as a result of symmetry, electric filed should vanish outside the circle). Solution to this problem can be written as an integral over the Bessel’s functions^17^. In cylindrical coordinates we will have:

13 $$\begin{aligned} \phi ^{II}(\rho ,\psi ,z)=\sum _{n}\int _{0}^{\infty }dke^{-kz}J_n(k\rho )\left( A_n(k)\sin n\psi +\bar{A}_n(k)\cos n\psi \right) ,\,\,\,z\ge 0, \end{aligned}$$

where the coefficients obey the following integral equations:

14 $$\begin{aligned} {\left\{ \begin{array}{ll} \int dk J_n(k\rho )A_n(k)=\phi _{\text {s}n}\,\,0\le \rho \le R,\\ \int dk J_n(k\rho )kA_n(k)=0\,\,R\le \rho \le \infty , \end{array}\right. }\,\,\, {\left\{ \begin{array}{ll} \int dk J_n(k\rho )\bar{A}_n(k)=\bar{\phi }_{\text {s}n}\,\,0\le \rho \le R,\\ \int dk J_n(k\rho )k\bar{A}_n(k)=0\,\,R\le \rho \le \infty . \end{array}\right. } \end{aligned}$$

here the surface potential is expanded as:

15 $$\begin{aligned} \phi _{\text {s}}(\rho ,\psi )=\sum _{n\ge 0}\left( \phi _{\text {s}n}(\rho )\sin n\psi +\bar{\phi }_{\text {s}n}(\rho )\cos n\psi \right) . \end{aligned}$$

Finally, the surface charge can be written as:

16 $$\begin{aligned} q^{II}(\rho ,\psi )=2\epsilon _0\sum _{n}\int _{0}^{\infty }dk kJ_n(k\rho )\left( A_n(k)\sin n\psi +\bar{A}_n(k)\cos n\psi \right) . \end{aligned}$$

Now, we are in a position to obtain the surface potential $\phi _{\text {s}}(\rho ,\psi )$. Along this task we need to solve the last equation in Eq. 9. Following the expansion presented in Eq. 15 for surface charge, we expand the charge distributions as:

17 $$\begin{aligned} q(\rho ,\psi )=\sum _{n\ge 0}\left( q_{n}(\rho )\sin n\psi +\bar{q}_{n}(\rho )\cos n\psi \right) . \end{aligned}$$

In terms of the above mentioned expansions for surface potential and surface charge, we will have:

$$\begin{aligned} \nabla _{\text {s}}^2\phi _\text {s}=\sum _n\left( \mathscr{D}^2\phi _{\text {s}n}\sin n\psi +\mathscr{D}^2\bar{\phi }_{\text {s}n}\cos n\psi \right) , \end{aligned}$$

and

$$\begin{aligned} \nabla _{\text {s}}\phi _\text {s}\cdot \nabla _\text {s}q= & {} \sum _{n,m} \left( \phi '_{\text {s}n}\sin n\psi +\bar{\phi }'_{\text {s}n}(\rho )\cos n\psi \right) \left( q'_{m}\sin m\psi +\bar{q}'_{m}\cos m\psi \right) \\&+\sum _{n,m}\frac{nm}{\rho ^2}\left( \phi _{\text {s}n}\cos n\psi -\bar{\phi }_{\text {s}n}(\rho )\sin n\psi \right) \left( q_{m}\cos m\psi -\bar{q}_{m}\sin m\psi \right) , \end{aligned}$$

where $$\mathscr{D}^2f=f''+\frac{1}{\rho }f'-\frac{n^2}{\rho ^2}f$$ and $$f'=df/d\rho$$.

Substituting the above expansions in the relation $q\nabla _{\text {s}}^2\phi _\text {s}=-\nabla _{\text {s}}\phi _\text {s}\cdot \nabla _\text {s}q$, we will arrive at hierarchal equations that relate different orders of $\phi _{\text {s}n}$ and $q_{\text {s}n}$ to each other. Such equations can help us to find the terms that appear in expansions given in Eqs. 15 and 17.

Following the above procedure, we can study in details the first two non-trivial terms. We will obtain the following results:

18 $$\begin{aligned}&\bar{q}_0=\bar{\phi }_{\text {s}0}=\bar{q}_1={\phi }_{\text {s}1}=0,\,\,q_1=\frac{8\epsilon _0E_{\text {ex}}}{\pi }\frac{\rho }{\sqrt{R^2-\rho ^2}}, \end{aligned}$$

and the potential $\bar{\phi }_{\text {s}1}$ satisfies:

$$\begin{aligned} \bar{\phi }''_{\text {s}1}+\frac{2R^2-\rho ^2}{\rho (R^2-\rho ^2)}\bar{\phi }'_{\text {s}1}-\frac{2}{\rho ^2}\bar{\phi }_{\text {s}1}=0,\,\,\,\,\bar{\phi }_{\text {s}1}(\rho =R)=-\frac{V_\text {in}}{2}. \end{aligned}$$

As it is seen, this equation needs some cares about the singularities at $\rho =R$ and $\rho =0$. We can analyze the above equation in two different regimes of $\rho \sim 0$ and $\rho \sim R$. The asymptotic equations read as:

19 $$\begin{aligned} {\left\{ \begin{array}{ll} \bar{\phi }''_{\text {s}1}+\frac{2}{\rho }\bar{\phi }'_{\text {s}1}-\frac{2}{\rho ^2}\bar{\phi }_{\text {s}1}=0,\,\,\,\,\,\,\,\,\,\rho \sim 0,\\ \bar{\phi }''_{\text {s}1}+\frac{1}{2(R-\rho )}\bar{\phi }'_{\text {s}1}-\frac{2}{R^2}\bar{\phi }_{\text {s}1}=0,\,\,\,\,\rho \sim R. \end{array}\right. } \end{aligned}$$

**Figure 2:**
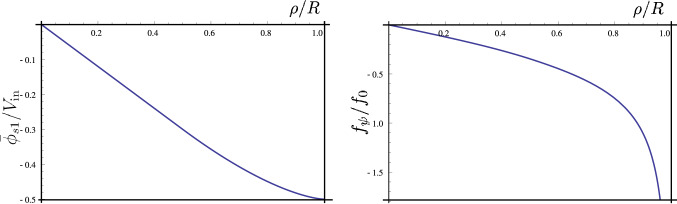
Left: Contribution to the surface potential $\bar{\phi }_{s1}/V_\text {in}$ is plotted in terms of $\rho /R$, distance from the center. Right: Angular component of force $f_\psi =\mathbf{f}_\text {b}\cdot {\hat{\psi }}$ scaled by $f_0=(8\epsilon _0/\pi )E_{\text {ex}}(V_{\text {in}}/R)$ is plotted in terms of $\rho /R$ at $\psi =\pi /2$

Considering the asymptotic solutions for the potential and using the fact that potential should be continuous everywhere, we can obtain a unique solution that is approximately valid everywhere. Solution to these equations read as:

20 $$\begin{aligned} {\left\{ \begin{array}{ll} \bar{\phi }_{\text {s}1}\sim -\frac{3}{5}{V_{\text {in}}}\frac{\rho }{R} ,\,\,\,\,\,\,\,\,\,\rho \le R/2,\\ \bar{\phi }_{\text {s}1}\sim \frac{V_{\text {in}}}{2}\left( -1+\frac{2^{5/2}}{5}(1-\rho /R)^{3/2}\right) ,\,\,\,\,\rho \ge R/2, \end{array}\right. } \end{aligned}$$

where we have demanded that the asymptotic solutions matches at $\rho =R/2$. In Fig. 2(left), potential distribution is plotted. As one can distinguish, deviation from the linear behavior get much impact very near to the edge of film.

## Flow field

Using the first order results for the surface distribution of charge and potential from previous part, we can obtain an analytic result for the body force that is acting on the fluid:

21 $$\begin{aligned} \mathbf{f}_{\text {b}}=\frac{8}{\pi }\epsilon _0E_{\text {ex}}\frac{V_{\text {in}}}{R}\frac{\rho }{\sqrt{R^2-\rho ^2}} {\left\{ \begin{array}{ll} \frac{3}{5}\sin \psi \cos \psi \,{\hat{\rho }}-\frac{3}{5}\sin ^2\psi \,{\hat{\psi }} ,\,\,\,\,\,\,\,\,\,\,\,\,\,\rho \le R/2,\\ \frac{3\sqrt{2}}{5}z^{\frac{1}{2}}\sin \psi \cos \psi \,{\hat{\rho }}-\frac{1}{2}\frac{R}{\rho }\left( 1-\frac{2^{\frac{2}{5}}}{5} z^{\frac{3}{2}}\right) \sin ^2\psi \,{\hat{\psi }},\,\,\,\,\rho \ge R/2, \end{array}\right. } \end{aligned}$$

where $$z=(1-\frac{\rho }{R})$$.

Before studying the results for the flow pattern, we note that the force field is characterized by a force scale given by $f_0=(8\epsilon _0/\pi )E_{\text {ex}}({V_{\text {in}}}/{R})$. This force density (force per unit area) when we divide it with the thickness of film $\lambda$, will give the driving force (force per unit volume) of the system. For a typical experiment, $f_0/\lambda \sim 10^{3}\text {N}/\text {m}^3$.

In attempting to use non-dimensional form of the Navier-Stokes equation for our problem, in addition to Reynolds number, another dimensionless number that measures the relative importance of body force to viscous force will appear. Denoting this dimensionless number by $\mathscr{F}$, we see that:

22 $$\begin{aligned} \mathscr{F}=\frac{\text {body force}}{\text {viscous force}}\sim \frac{[f_\text {b}]}{[\eta \nabla ^2 u]}\sim \frac{\epsilon _0E_\text {ex}V_\text {in}R}{\lambda \eta u}\sim 10^4. \end{aligned}$$

The force density given in Eq. 21 has both radial and polar components. As a result of no-slip boundary condition on the edge of frame, we expect to obtain a 2-dimensional rotational flow that is bounded by the circular frame. In Fig. 2(right), we have plotted the polar component of this force in terms of $\rho /R$, the dimensionless distance from the origin. The force field vanishes at the center but it diverges at the boundary.

To obtain the flow pattern associated to the above force field, taking into account both radial and polar components of the force, we proceed by numerically solving the Navier–Stokes equation. As long as the Reynolds number is not very high, we use a numerical approach based on finite elements method for solving laminar flow.

**Figure 3:**
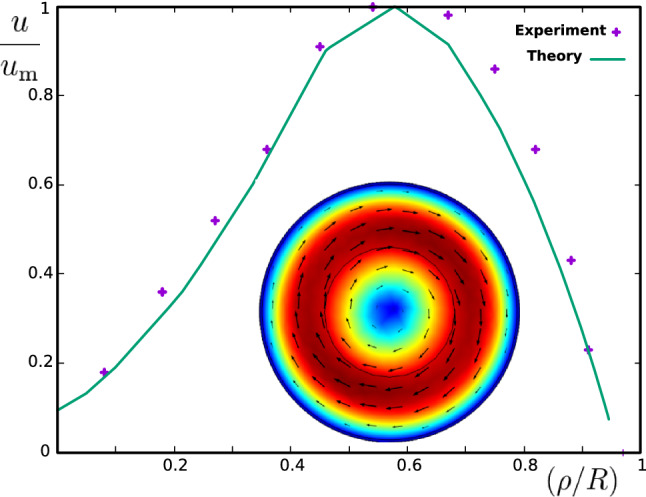
Experimental results (dots) and theoretical results (continuous graph and colored flow pattern) are shown. Speed of the fluid in terms of distance to the center of rotation is plotted. For a fixed distance, the speed is averaged over all angles. Both experimental (dots) and numerical (line) results are scaled by the maximum value of velocity $u_\text {m}$. Inset shows a snapshot of numerical solution to the flow equations taking into account the force field obtained from our theoretical results. Arrows show the direction of flow and colors, encode the strength of velocity. Other numerical values are given in the text

A typical example of the flow pattern obtained by numerical integration of the equations is shown in the inset of Fig. 3. Reflected from this figure, a circular flow pattern will be achieved at the steady state conditions. By averaging over angle, we have plotted the velocity at different radii. By scaling the velocity with its maximum value, we compare the results of our model with experimental data. From Fig. 3, a good agreement between our simplified model (continuous line) and the experimental values (dots) can be observed. As it is seen from our linearized theory, the maximum velocity appears to happen at a universal distance from center of rotation given by $\rho /R\sim 0.6$. This point is universal in a sense that it is controlled by two dimensionless numbers $\mathscr{F}$ and $\text {Re}$.

## Intuitive picture

Following the proposed model and the approximate solutions that we have provided in previous section, we can present a very simple picture of the underlying physics that is responsible for the rotation of fluid. At first glance, it seems that the Nonlinearity of the equations does not permit us to superimpose the effects of two electric fields. But our method of solution, demonstrates that at leading orders, how a simple picture for the physical mechanism can be constructed.

The external field ${E}_{\text {ex}}$, is responsible to separate positive and negative charges. As shown in Fig. 1(left), and consistent with our analytical result in Eq. 18, and for ${E}_{\text {ex}}>0$, positive (negative) charges are mainly accumulated near the edge of the liquid-film at $y=\ell /2$ ($y=-\ell /2$). The potential difference applied to the electrodes, will produce a surface electric field and this field enforce the fluid to move. For $V_{\text {in}}>0$, very near to the surface of liquid-film, the electric field will achieve a component that is roughly in ${\hat{x}}$ direction. This field will exert electric force to the accumulated charges. This electric body force will enforce the fluid to move in directions denoted by arrows in fig. 1(left). These local movements of fluid parcels produced in the positions of accumulated charges, will result a fluid circulation in the observed direction.

## Discussion

The geometry that we have considered is special in a sense that the external electric field is assumed to be perpendicular to the alignment of the internal voltage difference. For a more general case where the external field and the internal field have an angle $\Theta$ with respect to each other, we can simply expect to see a factor $\sin \Theta$ that scales all of our results. As a main approximation in our theory, we have considered only the first terms ($n=1$) in expansions in terms of $\sin (n\psi )$ and $\cos (n\psi )$. Total torque applied on the fluid is given by $\tau ={\hat{z}}\cdot \int d^2\mathbf{r}{} \mathbf{r}\times \mathbf{f}_\text {b}$ and this is the main quantity that controls the circular motion of the fluid. We think that higher order terms that have larger oscillations with angle $\psi$, will have less contribution into the total torque applied on the fluid.

A very important feature that is reflected from our theory is the universality of flow field. Direction of rotation is given simply with $\mathbf{E}_\text {ex}\times \mathbf{E}_\text {in}$ where, $\mathbf{E}_\text {in}$ shows the alignment of applied voltage difference (it points from higher to lower applied voltage). Interestingly the flow profile is also universal, in a sense that the flow is characterized with a single force amplitude $f_0$. Instead of geometrical dimension of the frame *R*, no other length scale enters into the results. This universal profile is reflected in Fig. 2, where angular velocity (scaled by its maximum value) has a universal profile in terms of radial distance scaled by *R*. Experimental results with different fluids and different electric forces^4^, show velocity profile that are in good agreement with the universal result have been obtained in this article.

In real films that has finite value of thickness, we should take into account the 3-dimensional structure of the film. In addition to $\lambda$, electric permittivity of the fluid should enters into the formulation. This may change the universal picture that we have provided for the flow structure.

In this work we have studied the conduction dominated regime where diffusion and convection of ions are neglected. A very important advantage of this regime is that the final results for the fluid flow are independent from the concentration of salt molecules. The whole system is electrically neutral but induced charges, can locally deviate the system from the neutrality condition. Electric charges in the system are mainly induced by electric fields. Irrelevance of salt concentration have been proved with experimental verifications.

It should be mentioned that in some previous works^13^, the conduction current that appeared in Eq. 5 is replaced simply by $-\sigma \nabla _\text {s}\phi _\text {s}$ where $\sigma$ stands for the bulk conductivity of the fluid. Such assumption neglects the contribution from spatial variation of the charge in the current. As a result of such approximation, the nonlinear term in Eq. 9 will be dropped and everywhere on the film, the surface potential will change linearly (linear with respect to $\rho$).

In summary, we have provided a theoretical model for liquid-film motor in the regime of negligible thickness. At the linear order, this theory provides a universal flow profile for the liquid. For a real film with finite thickness, we are working to see how such results are universal or not.

## References

[1] Squires TM, Quake SR (2005). Microfluidics: Fluid physics at the nanoliter scale. Rev. Mod. Phys..

[2] Bruus H (2008). Theoretical Microfluidics.

[3] Amjadi A, Shirsavar R, Radja NH, Ejtehadi M (2009). A liquid film motor. Microfluid. Nanofluid..

[4] Shirsavar R, Amjadi A, Tonddast-Navaei A, Ejtehadi M (2011). Electrically rotating suspended films of polar liquids. Exp. Fluids.

[5] Faetti S, Fronzoni L, Rolla P (1983). Static and dynamic behavior of the vortex-electrohydrodynamic instability in freely suspended layers of nematic liquid crystals. J. Chem. Phys..

[6] Stannarius R, Bohley C, Eremin A (2006). Vortex flow in freestanding smectic films driven by elastic relaxation of the c director. Phys. Rev. Lett..

[7] Zakharov A, Sliwa I (2015). Squeezing-out dynamics in free-standing smectic films. J. Chem. Phys..

[8] Zakharov A, Vakulenko A (2015). Orientational relaxation in free-standing smectic c film driven by rotating circular frame. J. Chem. Phys..

[9] Morris SW, de Bruyn JR, May A (1990). Electroconvection and pattern formation in a suspended smectic film. Phys. Rev. Lett..

[10] Zhang J, Sheng L, Liu J (2014). Synthetically chemical-electrical mechanism for controlling large scale reversible deformation of liquid metal objects. Sci. Rep..

[11] Sheng L, Zhang J, Liu J (2014). Diverse transformations of liquid metals between different morphologies. Adv. Mater..

[12] Nasiri M, Shirsavar R, Saghaei T, Ramos A (2015). Simulation of liquid film motor: A charge induction mechanism. Microfluid. Nanofluid..

[13] Feiz M, Namin R, Amjadi A (2015). Theory of the liquid film motor. Phys. Rev. E.

[14] Shiryaeva E, Vladimirov V, Zhukov MY (2009). Theory of rotating electrohydrodynamic flows in a liquid film. Phys. Rev. E.

[15] Deyirmenjian V, Daya ZA, Morris SW (1997). Weakly nonlinear analysis of electroconvection in a suspended fluid film. Phys. Rev. E.

[16] Smythe WB (1988). Static and Dynamic Electricity.

[17] Jackson JD (1999). Classical Electrodynamics.

